# Virus sequencing performance during the SARS-CoV-2 pandemic: a retrospective analysis of data from multiple rounds of external quality assessment in Austria

**DOI:** 10.3389/fmolb.2024.1327699

**Published:** 2024-02-05

**Authors:** Jeremy V. Camp, Elisabeth Puchhammer-Stöckl, Stephan W. Aberle, Christoph Buchta

**Affiliations:** ^1^ Center for Virology, Medical University of Vienna, Vienna, Austria; ^2^ Austrian Association for Quality Assurance and Standardization of Medical and Diagnostic Tests (ÖQUASTA), Vienna, Austria

**Keywords:** SARS-CoV-2, next-generation sequencing, diagnostic laboratories, Austria, COVID-19 diagnostic testing

## Abstract

**Introduction:** A notable feature of the 2019 coronavirus disease (COVID-19) pandemic was the widespread use of whole genome sequencing (WGS) to monitor severe acute respiratory syndrome coronavirus 2 (SARS-CoV-2) infections. Countries around the world relied on sequencing and other forms of variant detection to perform contact tracing and monitor changes in the virus genome, in the hopes that epidemic waves caused by variants would be detected and managed earlier. As sequencing was encouraged and rewarded by the government in Austria, but represented a new technicque for many laboratories, we designed an external quality assessment (EQA) scheme to monitor the accuracy of WGS and assist laboratories in validating their methods.

**Methods:** We implemented SARS-CoV-2 WGS EQAs in Austria and report the results from 7 participants over 5 rounds from February 2021 until June 2023. The participants received sample material, sequenced genomes with routine methods, and provided the sequences as well as information about mutations and lineages. Participants were evaluated on the completeness and accuracy of the submitted sequence and the ability to analyze and interpret sequencing data.

**Results:** The results indicate that performance was excellent with few exceptions, and these exceptions showed improvement over time. We extend our findings to infer that most publicly available sequences are accurate within ≤1 nucleotide, somewhat randomly distributed through the genome.

**Conclusion:** WGS continues to be used for SARS-CoV-2 surveillance, and will likely be instrumental in future outbreak scenarios. We identified hurdles in building next-generation sequencing capacity in diagnostic laboratories. EQAs will help individual laboratories maintain high quality next-generation sequencing output, and strengthen variant monitoring and molecular epidemiology efforts.

## 1 Introduction

Within 1 month of the first detection of the novel coronavirus in December 2019, the dissemination of the virus sequence, as well as a publicly available database of similar sequences, allowed the development of the first diagnostic tests based on RT-qPCR for SARS-CoV-2 (Corman et al., 2020). A month later, in February 2020, reference laboratories in Europe (Reusken et al., 2020) and elsewhere were prepared to detect the virus with validated protocols. The new tools and kits prepared and sold by commercial entities initially were allowed emergency use certification as *in vitro* diagnostic (IVD) tests, pending more rigorous performance analysis. However, as the basic techniques for virus genome detection were already widespread in diagnostic laboratories, it was shown through external quality assessments (EQA) around the world that initial overall performance was high, with relatively few false negative results and almost no false positive results (Görzer et al., 2020). EQA schemes on SARS-CoV-2 genome detection served to inform public health authorities about the quality of epidemic data generated by diagnostic laboratories, but, equally importantly, informed participants about their performance relative to other labs so that they may identify areas for improvement (Buchta et al., 2023a). This is a critical process when new assays or techniques are implemented, as seen during the COVID-19 pandemic when the performance of a given assay was unknown and their implementation unfamiliar to some diagnostic laboratories (Buchta et al., 2023b).

Beyond mass testing to identify virus-positive individuals, the COVID-19 pandemic provided additional challenges for public health, namely tracking the emergence of novel viral variants. This task largely fell on the same diagnostic laboratories performing genome detection assays. Some laboratories opted to rely on more familiar assay formats (RT-qPCR and melting curve assays) to identify specific mutations characteristic of new lineages. While the assay format was familiar, the correct interpretation of the results was complex and often led to ambiguous results (Camp et al., 2021; Buchta et al., 2022a). Alternatively, some laboratories rapidly implemented whole genome sequencing (WGS) to characterize the viruses from patient samples. This expanding sequencing capacity was realized in laboratories across the globe, particularly in resource-poor countries where sequencing may not have been previously available. The rapid expansion of sequencing capacity was facilitated, in part, by i) the development and reliability of a whole genome sequencing strategy (Quick et al., 2017), ii) the availability of affordable 2nd and 3^rd^ generation sequencing devices, and iii) the development and availability of bioinformatic pipelines. Together, generating consensus sequences of a virus from next-generation sequencing (NGS) platforms became achievable for the average clinical laboratorian. Online sequence databases began accumulating sequences, and, as the virus changed, governments encouraged–or even required–diagnostic and reference laboratories to provide sequences to these databases to track variants.

The result was that, at of the time writing, over 16 million sequences of SARS-CoV-2 have been uploaded to the Global Initiative on Sharing All Influenza Data (“GISAID”, a database that emerged during the COVID-19 pandemic as a repository of SARS-CoV-2 sequences (Shu and McCauley, 2017)). Such comprehensive genetic coverage of a single virus has never been achieved before, and the compendium of genetic information has driven detailed analyses of the global evolution of SARS-CoV-2. However, the quality and/or accuracy of individual sequences is unknown. In contrast to virus genome detection assays, there were relatively few commercial options for preparing samples for SARS-CoV-2 WGS (First NGS, 2020), and these approaches were empirically tested, which could assist laboratories in selecting and validating suitable sequencing approaches (Charre et al., 2020; Liu et al., 2021). However, benchmarking bioinformatics pipelines for NGS data remains an issue in multiple fields, even when wet lab techniques in clinical diagnostics are established (Angers-Loustau et al., 2018; SoRelle et al., 2020; de Vries et al., 2021; Krishnan et al., 2021). Well into the COVID-19 pandemic, it was clear to some experts that deficiencies existed due to a lack of familiarity/competency in the bioinformatics analyses required for the producing quality data from NGS platforms, and this was hindering the utility of genomic surveillance (Hanahoe et al., 2021; Hodcroft et al., 2021).

The use of NGS in diagnostics has become more established in other fields. For example, there exist CE-certified *in vitro* diagnostic (IVD) kits for preparing samples from patients to identify germline or somatic-mutation related diseases, and these can be used on CE-IVD NGS platforms (e.g., Illumina MiSeqDx or Ion Torrent Genexus Dx). More recently, FDA-approved and CE-IVD tests can process patient samples (tissues or liquid biopsies) to detect and diagnose cancer on NGS platforms, as part of personalized medicine approaches (Jennings et al., 2017). Although NGS has been widely adopted by microbiology laboratories to complement diagnosis, identify microbial resistance, or profile the microbiome, the process towards validation of these techniques for routine use in diagnostics is less clear (Rossen et al., 2018). Similarly, there has been limited use of sequencing in routine clinical virology prior to the SARS-CoV-2 epidemic, mostly Sanger-sequencing relatively short nucleotide sequences for the purposes of antiviral resistance testing (e.g., HIV or Hepatitis B virus), and mostly limited to expert reference laboratories. The performances of some of these assays/techniques had been evaluated via EQA (Germer et al., 2013; Lee et al., 2020; Parkin et al., 2020), but these studies show that many laboratories preferred alternative methods for genotyping that did not require sequencing. At the time of writing, we know of only one CE-IVD assay for the sequencing of SARS-CoV-2; this status was only recently achieved and only under emergency use only regulations (Illumina COVIDSeq).

The benefits of virus genomic surveillance were evident prior to the COVID-19 pandemic (Dudas et al., 2017; Faria et al., 2017; Oude Munnink et al., 2021), were implemented in various countries during the COVID-19 pandemic (Oude Munnink et al., 2020), and will become essential to monitoring and controlling future epidemics. As SARS-CoV-2 variant monitoring was seen as an integral part of managing the pandemic, and drove public health policy decisions, the Austrian government provided a bounty on sequences submitted to public databases. Therefore, monitoring the performance of laboratories reporting sequencing data based on NGS techniques and ensuring their accuracy is of high importance. Having previously designed EQA schemes for SARS-CoV-2 virome detection (Buchta et al., 2023c), we sought to design an EQA scheme to test the performance of laboratories performing whole genome sequencing. Additionally, analyzing concordance between laboratories could provide some insight into the accuracy of publicly available sequencing data. As EQA also serves the function of providing direct feedback to participants, we tracked performance of some laboratories over several rounds, to see if performance improved.

However, to our knowledge there have been only two published EQA schemes to evaluate consensus sequences generated by virus whole genome sequencing, and there were none at the time when we implemented our scheme (Lau et al., 2022; Wegner et al., 2022). The goal of this project was to implement an EQA scheme to assess laboratories performing whole genome sequencing of SARS-CoV-2 in Austria. We report the results of a SARS-CoV-2 WGS EQA scheme over five rounds from February 2021 until June 2023, analyzing performance in terms of sequence accuracy, and the ability to interpret viral genomic data. We note changes in performance over time, and we discuss changes in the EQA scheme over time to highlight the difficulties that we experienced in designing such a scheme, particularly in comparison to other published schemes.

## 2 Materials and methods

### 2.1 Sample preparation and scheme organization

The EQA schemes in Austria were administered by the Austrian Association for Quality Assurance and Standardization of Medical and Diagnostic Tests (ÖQUASTA), providing the technical infrastructure associated with coordinating participant enrollment, distribution of sample materials, and collecting results. The Center for Virology at the Medical University of Vienna provided expertise in selecting and validating test samples as well as analyzing the reported results.

The EQA schemes for SARS-CoV-2 whole genome sequencing occurred in February 2021 July 2021, February 2022 October 2022, and May 2023 (Table 1). Enrollments were confirmed when the participant provided information on i) sequencing protocol including reagents or kits and specific primer panels; ii) sequencing platform; and iii) basics of bioinformatics pipeline used in analysis. Participants were mailed a panel of 4–5 samples mostly derived from residual material (oropharyngeal swab) received as part of routine diagnostic testing or occasionally plaque-purified virus isolate (Vero cells), and therefore no specific ethical approval was required. Samples were prepared by dilution in physiological saline, or (for the 4th and 5th rounds) in RNAlater^®^ (Thermo Scientific). SARS-CoV-2 sequences from the samples were characterized initially by the reference laboratory as part of routine surveillance. The prepared panel was quantified by RT-qPCR targeting the E gene, and sequenced again. To ensure homogeneity of the prepared sample panel, each sample was sequenced 2–3 times in total by the reference laboratory prior to shipping; and at least one of those was after mimicking extreme shipping conditions (stored for 1 week at room temperature) to assess the stability of the samples. At least one of these quality control sequencing runs was performed on an Illumina MiSeq, and one was performed on a MinION Mk1c for the 3rd, 4th, and 5th rounds.

**TABLE 1:** SARS-CoV-2 whole genome sequencing EQA schemes in Austria.

Round	Date	Sample	GISAID EPI_ISL_#	Mean Ct	Lineage
1	Feb 2021	hCoV-19/Austria/CeMM2633/2021	934568	29.5	B.1.1.7
		hCoV-19/Austria/MUW_1375876/2021	1191133	31.1	B.1.1.7
		hCoV-19/Austria/CeMM3247/2021	1008244	27.1	B.1.351
		hCoV-19/Austria/MUW_1320744/2021	913069	29.6	B.1.177
		hCoV-19/Austria/MUW_1358160/2021	913078	30.3	B.1.258
2	July 2021	hCoV-19/Austria/MUW_9133135702/2021	3144944	22.0	B.1.1.7 + S:E484K
		hCoV-19/Austria/MUW_1413581/2021	3144945	23.0	B.1.1.318
		hCoV-19/Austria/MUW_1379219/2021	1191134	29.5	B.1.351
		hCoV-19/Austria/MUW_1420272/2021	3144946	23.8	B.1.617.2
		hCoV-19/Austria/MUW_204840628007/2021	3144947	25.6	P.1
3	Feb 2022	hCoV-19/Austria/CeMM21006/2021	7798629	23.6	B.1.617.2
		hCoV-19/Austria/CeMM21823/2021	9011257	26.6	BA.1.1
		hCoV-19/Austria/MUW_1481609/2022	n/a	24.6	BA.2
		hCoV-19/Austria/CeMM20996/2021	7798619	24.6	AY.34
4	Oct 2022	hCoV-19/Austria/MUW_1513521/2022	13328434	22.0	BA.2
		hCoV-19/Austria/MUW_1513519/2022^a^	13328433	23.0	BA.2
		hCoV-19/Austria/MUW_1511131p2/2022^b^	15982848	18.0	BA.5.3
		hCoV-19/Austria/MUW_1511131p2/2022^b^	15982848	20.0	BA.5.3
5	May 2023	hCoV-19/Austria/MUW_1567739/2023	16006120	25.0	BF.11.3
		hCoV-19/Austria/MUW_1572048/2023	n/a	23.0	BQ.1.1.49
		Negative (HeLa cell culture supernatant)	n/a	n/a	n/a
		hCoV-19/Austria/MUW1584021/2023	17062380	20.0	XBB.1.5.12
		hCoV-19/Austria/MUW_1586996/2023	17247178	25.0	DB.1

^a^ Sample with minor variants.

^b^ Duplicate samples at two dilutions.

Samples representing contemporary circulating variants were selected each round (Table 1). Specimens with high estimated viral genome copy number were preferred, as dilutions were required to prepare the material for distribution. Mostly, “non-challenging” samples were selected, however some “challenging” or educational samples were included in later rounds. In round four (total four samples), a sample with minor variants (>30%) was included to test participant interpretation. In round four, two samples were the same isolate at two different dilutions (mean *Ct* values of 18 and 20) to test within-lab reproducibility. In round five, a sample negative for SARS-CoV-2 genome was included to test participant quality control measures and cross-contamination.

### 2.2 Reporting results

Participants were provided information about sample preparation, and instructed to sequence the panel using normal/routine protocols. Reporting could be carried out using an online system, however, the participants were provided a report form to fill out and (e-)mail or fax. Although the report format changed slightly as the scheme evolved, the requested results were the same in each round, designed to test two main competencies:

i. The ability to generate an accurate sequence.
ii. The ability to manage and interpret sequence data.

### 2.3 Technical evaluation of sequence accuracy

For the first two rounds, for each sample, the participants were requested to provide all nucleotide differences in comparison to the reference strain (the NCBI Reference Sequence “Wuhan-Hu1”, GenBank accession number NC_045512.2). For rounds 3, 4, and 5, the participants were requested to submit sequence results in fastn format. Completeness was the percent of the genome reported as a nucleotide (A/T/C/G) or ambiguous symbol (K/M/R/S/W/Y). Participants were not penalized for missing genetic data (N’s). In order to reduce bias, the consensus sequence from all submitted sequences (or the reported inferred sequence in rounds 1 and 2) and at least two sequences generated by the reference laboratory was considered the “true” sequence. Sequence accuracy was assessed by determining the number of differences in the submitted sequence (or the reported inferred sequence in rounds 1 and 2) compared to this consensus sequence. Mutations, insertions, and deletions compared to the consensus sequence were counted, and the number of differences was the Accuracy Score, where a higher value is worse. For the purposes of evaluation (pass/fail), participants passed if fewer than six differences were found relative to the consensus.

### 2.4 Technical evaluation of sequence interpretation

The ability to manage and interpret sequence data was assessed in two ways, by asking participants to characterize each sample based on its sequence. Therefore, the following “interpretation” results were evaluated based on each individual (submitted) sequence, and not on the “true”/consensus sequence that was used for the Accuracy Score.

#### 2.4.1 Lineage interpretation

For each sample, the participants were requested to provide a Pangolin lineage assignment for their sequence (O'Toole et al., 2021). The submitted lineages were evaluated based on correctness (Pass/Fail/Can be improved) based on an independent assessment of lineage from the submitted fastn file.

#### 2.4.2 Mutation reporting

For the 3^rd^, 4^th^, and 5^th^ rounds, the participants were required to report amino acid mutations (and indels) with respect to the reference sequence (“Wuhan-Hu-1”, NC_045512) in the spike protein only. This was done mostly out of practicality, given the increasing number of mutations in the virus genome, but also to continue testing the ability of the participant to interpret sequence data. Similar to the Accuracy Score, a Self-Reported Mutation Score was recorded as the number of differences between the submitted substitutions in the inferred spike protein and the independently-determined substitutions.

### 2.5 Statistical analysis

We used descriptive statistics to describe laboratory performance with respect to four measured results: completeness, accuracy, self-reported mutation score, and pangolin lineage assignment. We note the number of laboratories passing all samples, as well as the number of samples successfully sequenced as a function of their virus load (estimated average *C*_*t*_ value). The participation was relatively low (minimum 3 participants/round, maximum 8), with participation varying haphazardly. Furthermore, no two laboratories reported using exactly the same protocol. Therefore, statistical power was too low to perform robust statistical comparisons (e.g., if there was a relationship between accuracy and specific platforms or sample preparation kits).

## 3 Results

### 3.1 Participants, platforms, protocols, and pipelines

Nine laboratories were registered over the course of five ring tests, two of which performed Sanger sequencing of a partial sequence, and were not considered in this manuscript (*n* = 7 participants). Participation varied within a given round between three and seven participants per round, with the nine laboratories participating in at least two rounds (Table 2). Incomplete results were reported by some participants and are considered missing data here. For example, one participant only submitted fastn sequences in one of the three rounds where it was specifically requested. Enrollment in the EQA scheme was voluntary, and the identity of the labs kept anonymous for evaluation purposes. However, we note that none of the laboratories performing virus genome surveillance in an official, government-sanctioned capacity participated, and participants were comprised of varying laboratory types (medical diagnostic and nonmedical; established clinical laboratories and newly implemented SARS-CoV-2-dedicated laboratories).

**TABLE 2:** Participation and methods in SARS-CoV-2 whole genome sequencing EQA schemes in Austria.

ID	Sample preparation (primers if known)	Platform	Bioinformatics	Rounds
A	AmpliSeq	Illumina MiniSeq	DRAGEN Illumina	2, 3, 4
B	QIAseq	Illumina NextSeq	QIAGEN cov2insight	1, 2, 3
C	AmpliSeq	Ion Torrent	Ion Torrent suite	2, 3, 4, 5
D	AmpliSeq	Ion Torrent	Ion Torrent suite	1, 2, 3
E	NEBNext (ARTIC)	Illumina NextSeq	[Not reported]	2, 3, 4
F	NEBNext (ARTIC)	Illumina MiSeq and MinION Mk1c	In house	1, 2, 3, 4, 5
G	NEBNext (ARTIC)	Illumina MiSeq	nf-core/Viralrecon	3, 5

Four laboratories used exclusively Illumina platforms (three for three rounds and one for two rounds), and two used exclusively Ion Torrent platforms (for four and two rounds); one participant used Illumina for three rounds, and an MinION Mk1c (Oxford Nanopore Technologies) for two rounds.

Sample preparation consisted of targeted amplification using tiled amplicon approaches for all participants in all rounds, with three laboratories using AmpliSeq (two Ion AmpliSeq, ThermoFisher; one AmpliSeq for Illumina, Illumina), two using QIAseq (QIAGEN), and three using NEBNext^®^ chemistry (New England Biolabs) prepared kits (*N.B.* two laboratories switched sample preparation methods during the course of the ring tests). Only one laboratory used two platforms during the five rounds: in-house reagents for amplification using ARTIC network primers followed by either the Nextera XT (Illumina) tagmentation kit or the NEBNext^®^ (New England Biolabs) ligation barcoding kits on the Illumina platform, or the native barcoding kit (EXP-NBD104) with v9.4.1 chemistry (Oxford Nanopore Technologies) for multiplexing and library prep on the MinION platform. Those using the NEBNext chemistry or in-house procedures reported using the ARTIC network primers (v3 or v4.1) (Quick, 2020; Tyson et al., 2020), and one reported using VarSkip short primer set (v1 and v2, New England Biolabs).

In terms of bioinformatics pipelines to generate the consensus sequences, both laboratories using Ion AmpliSeq (ThermoFisher) kits and Ion Torrent platforms used the Ion Torrent suite software for data analysis. Similarly, the participant using the AmpliSeq reagents for Illumina platforms used the Illumina DRAGEN software, and the participant using the QIAseq used unspecified QIAGEN software. One laboratory used the Viralrecon Nextflow pipeline (from nf-core, https://nf-co.re), and one laboratory used an in-house pipeline for Illumina (fastp, minimap2, samtools, and iVar) and porechop with medaka_consensus (Oxford Nanopore Technologies) for sequencing on the MinION platform.

### 3.2 Sequence accuracy and completeness

**FIGURE 1:**
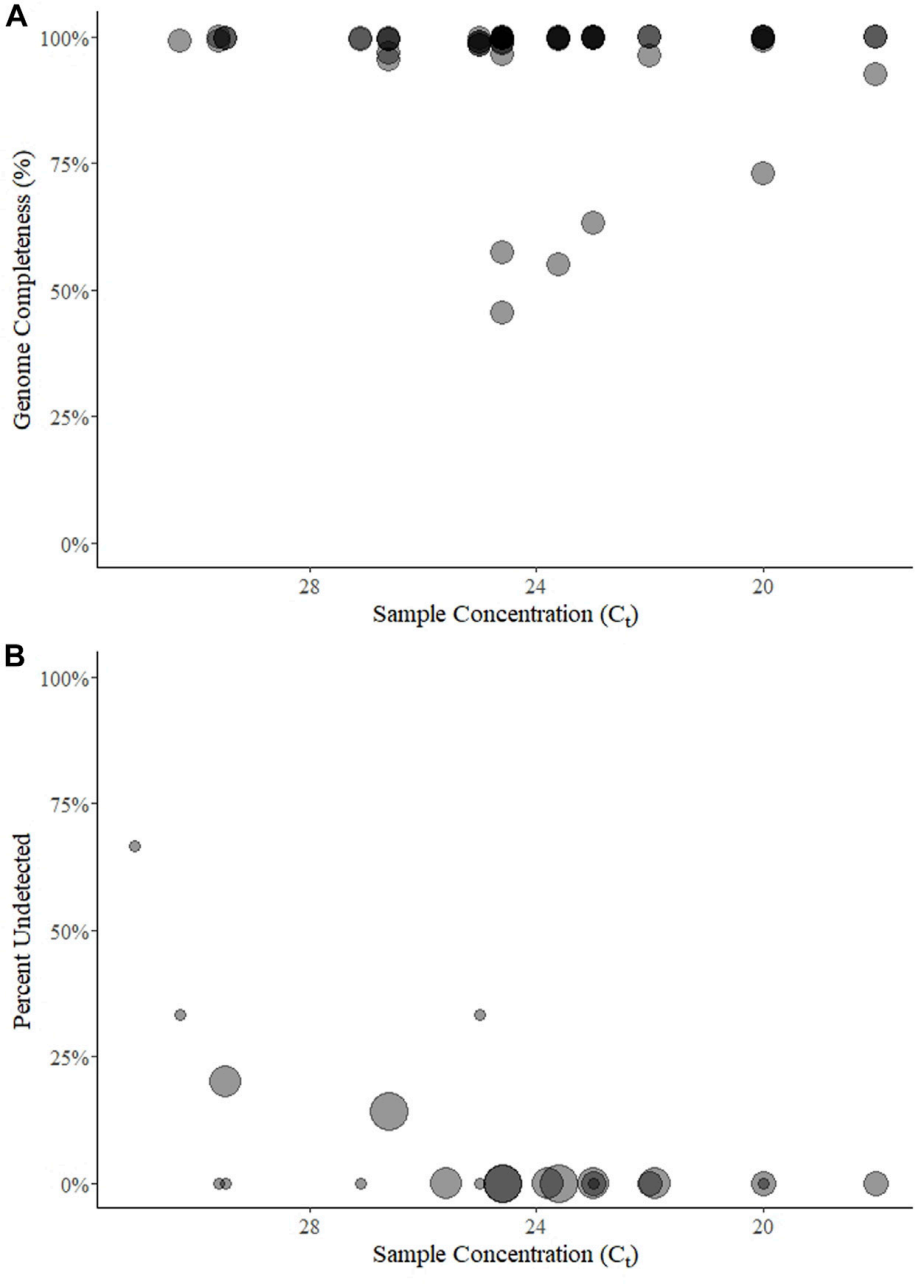
Genome completeness **(A)** and percent of samples undetected **(B)** over five rounds of a SARS-CoV-2 sequencing EQA in Austria. Points for genome completeness **(A)** are shown over approximate sample concentration (estimated by *C*_*t*_ value) from each submitted result (*n* = 54). The size of points in percent of results undetected **(B)** is relative to the number of participants (between 3 and 7) that submitted a result for a given sample (*n* = 22). In both panels the points are transparent gray and appear darker when overlapping.

In total, 99 test results were submitted over the five rounds (15-25-28-16-15 per round). Three of these (round 5) were from a sample that contained no SARS-CoV-2, and one of the three participants submitted a partial genome from this sample. From the 96 remaining test results, genome completeness was estimated from 53 sample-results. Completeness could only be taken from Rounds 1 and 2 if the participant specifically reported it (two did in round 1, *n* = 7, but none did in round 2); fastn files were never reported by a single participant in rounds 3 and 4 (*n* = 14); and if the sample was not sequenced it was considered “missing” and not 0%. Nonetheless, the mean genome completeness was 95% (range 45%–100%, sd = 12%) (Figure 1A). Only six results had a completeness <95%, (range 45%–92%) and were from a single participant on two consecutive rounds (3^rd^ and 4^th^) for samples with *C*_*t*_ values in a range from 23.6 to 31.3. Ignoring these outliers, the mean completeness was 99% (median 100%, s.d. = 1%).

Among the 96 possible test results for SARS-CoV-2-positive samples, 6 (6%) were “not detected” and came from five unique samples with mean E gene *C*_*t*_ values of 25 (*n* = 1 of three submitted results for that round), 26.6 (1 of 7), 29.5 (2 of 5), 30.3 (1 of 3), and 31.1 (2 of 3) (Figure 1B). Three of the undetected results came from one lab in two rounds (using the QIAseq protocol on an Illumina NextSeq) for samples with *C*_*t*_ ≥ 29.5.

**FIGURE 2:**
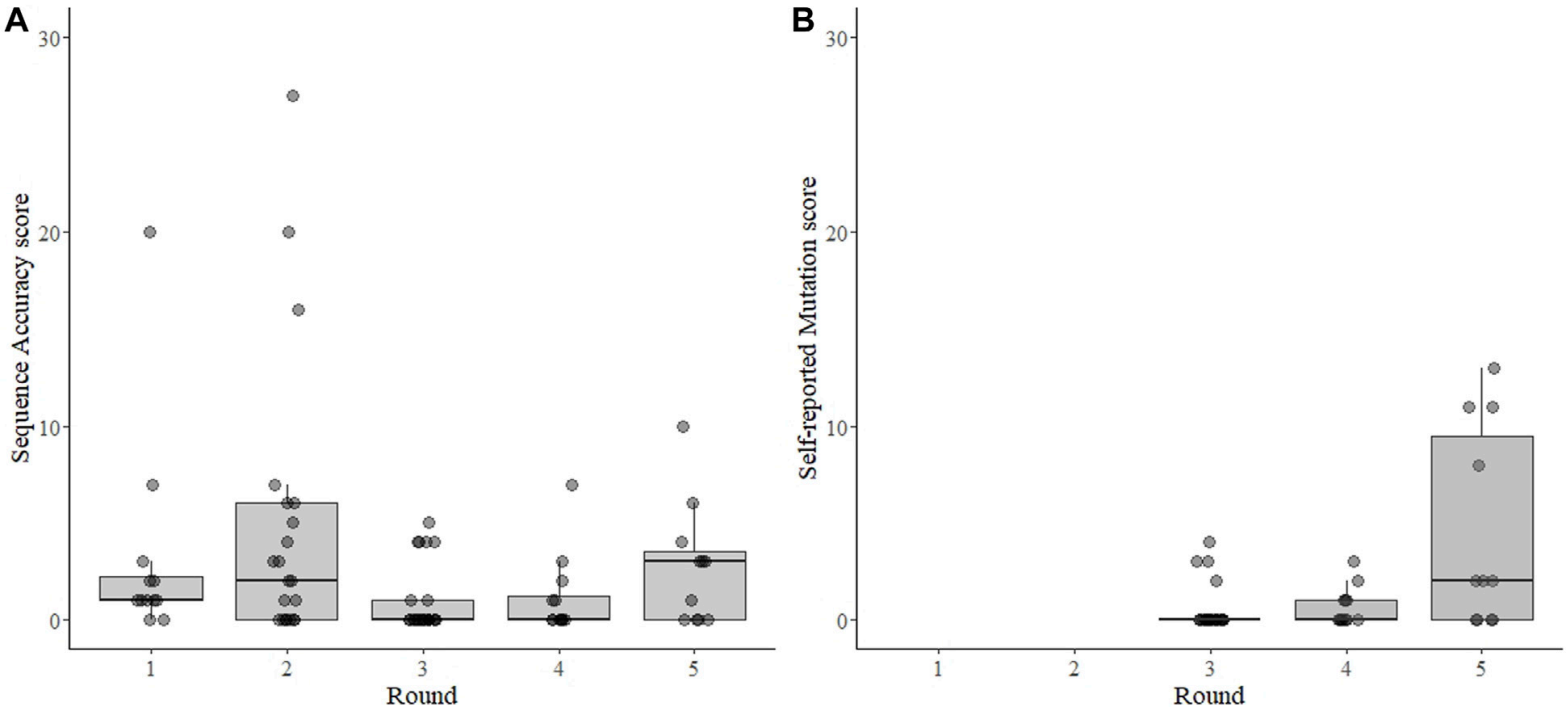
Sequence accuracy scores for SARS-CoV-2 sequencing EQAs **(A)** and scoring for self-reported mutation in the spike gene **(B)**. Each point represents the result from each sample submitted by each participant for each of five rounds of EQAs with boxes around the interquartile range and a thick horizontal line indicating the median value. The sequence accuracy score **(A)** is the number of differences from the consensus sequence of all laboratories for each sample. The accuracy score was independently verified for the 3^rd^, 4^th^, and 5^th^ rounds, but were inferred from submitted mutations for the 1^st^ and 2^nd^ rounds as no fastn files were requested during those two rounds. The self-reported mutation score **(B)** is a count of the differences between the non-synonymous amino acid mutations in the spike protein reported by the participant and an independent analysis of the mutations by the evaluator, which was calculated for the 3^rd^, 4^th^, and 5^th^ rounds. In both panels the points are transparent gray and appear darker when overlapping.

The sequence Accuracy Score could not be calculated from 17 sequence results (6 times when the sample was reported “virus not detected” or 11 times when the participant did not submit the requested results, as described above). From the remaining 79 test results, the mean Accuracy Score was 2.6 and the median was 1.0 (range 0–27, sd = 4.8) (Figure 2A). The distribution was skewed, with 48 (61%) results having one or fewer differences from the consensus and 36 results (45%) having no differences from the consensus. Only 11 results (14%) were categorized as failing (>5 differences), five of which came from one participant that participated in three rounds for a total of 13 tests (Supplementary Figure S1).

**FIGURE 3:**
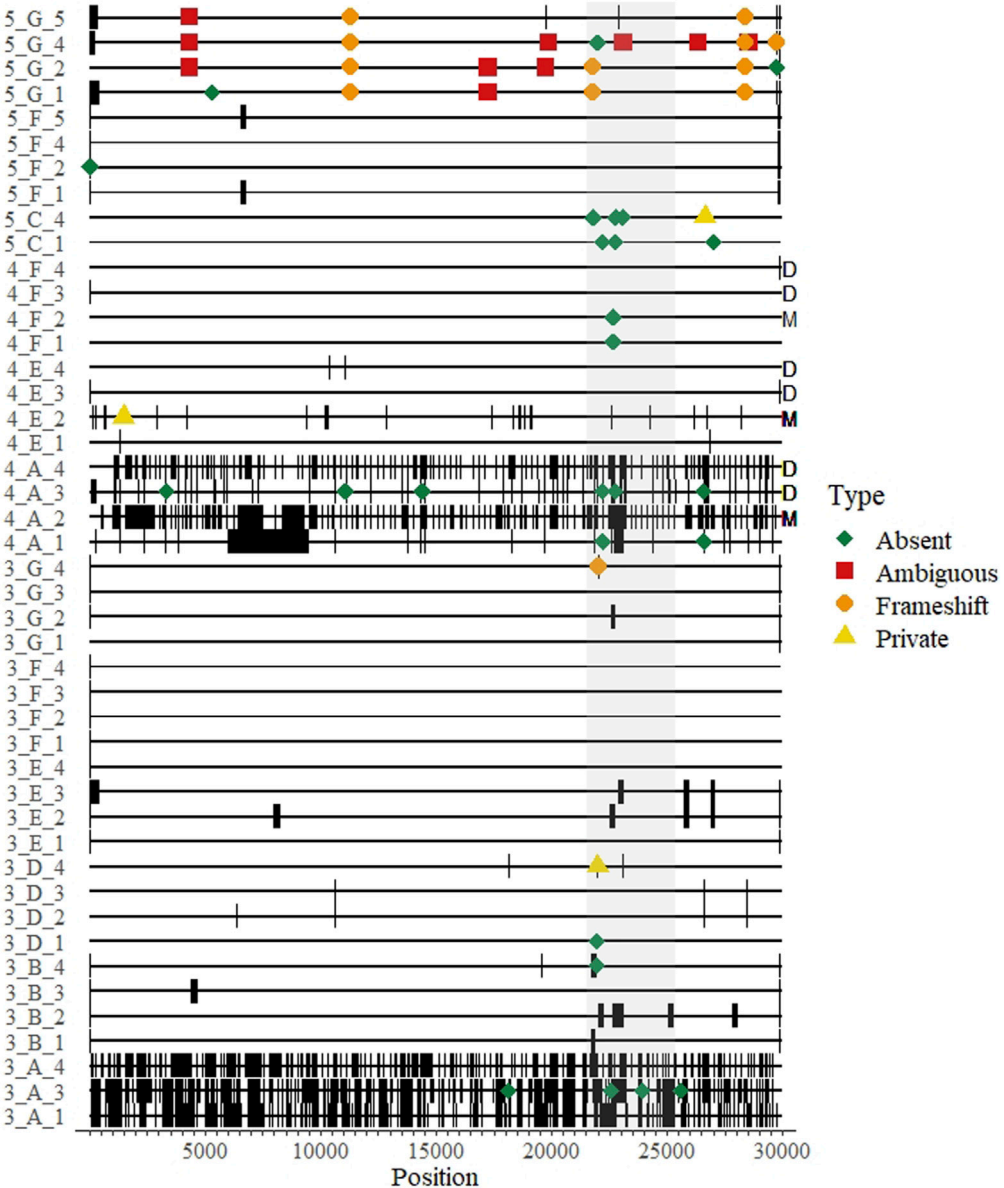
A genomic map of all whole genome sequences submitted over three rounds of a SARS-CoV-2 sequencing EQA in Austria missing data are thick black bands. Differences from the consensus and their approximate location on the genome are indicated by colored shapes: red squares indicate ambiguous nucleotides were called; orange circles represent indels that created a frameshift within an open reading frame; yellow triangles indicate a “private” difference from the consensus sequence that was not in the reference sequence (Wuhan-Hu-1, NC_045512); and green diamonds indicate mutation in the consensus sequence relative to the reference sequence (Wuhan-Hu1) that was not detected (“absent”). The sequence maps are grouped and labeled by round of EQA, laboratory letter as in Table 2, and Sample Number (Round_Lab_Sample) on the left. Letters on the right indicate results from duplicated samples (“D”) or the sample with minor variants (“M”).

Over three rounds where accuracy could be independently validated (3^rd^–5^th^ when fastn sequences were submitted), there were a total of 56 differences in submitted sequences compared to the corresponding consensus (Figure 3). Of these, 10 (18%) were ambiguous nucleotides (A/K/M/R/S/Y), and were not counted towards the Accuracy Score. Sequences with ambiguous nucleotide codes were all submitted by the same laboratory in the same round.

The remaining 46 differences comprised the Accuracy Scores, and could be classified into two main categories: frameshift mutations (indels, *n* = 12) and unique mutations (*n* = 34). As each submitted sample genome was compared to the consensus, the unique mutations could either be classified as “absent” (i.e., a mutation in the consensus relative to the reference strain that was *not* present in the submitted sequence) or “private” (i.e., a mutation present in the submitted sequence relative to the reference strain that was *not* present in the consensus).

There were twelve (of 46 = 26%) recorded instances where a participant submitted an indel in their sequence that would have resulted in a frameshift mutation. Eleven (92%) of the frameshifts were from a single lab in the fifth round that correctly identified deleted regions, but reported them to be shorter than expected (e.g., a 9 nt deletion in ORF1a was reported as a 4 nt deletion). The majority (34/46 = 74%) of the errors were unique mutations, three of them were private mutations and 31 (67% of all differences) were “absent” mutations. Of note, 20 of the 56 differences (36%) were in the spike protein open reading frame (Figure 3).

One sample in round four contained minor variants at five sites at a level of 11%–37% of the called bases per site, as determined by the initial sequencing done by the reference laboratory (Supplementary Table). Three laboratories participated in this round, but one produced no sequence data for four of the five sites (Figure 3, “M”). The majority variant was detected by all laboratories at three sites, where the nucleotide composition was determined to be 63%, 85% and 89% of the reads. The minor variant was called by all laboratories except the reference laboratory once, where the minor variant was 34% of the reads; and all but one laboratory detected the majority variant at a site where the minor variant was 30%. Also in round four, two of the four laboratories reported exactly the same sequence for both replicated isolates (one even suggested in the optional notes that they were the same sample and not a cross-contamination) (Figure 3, “D”). One laboratory had different sequence scores and mutation scores for this sample (7 vs. 3, respectively for the *C*_*t*_ 18 the *C*_*t*_ 20 samples) (Figure 3, “4_A_3” and “4_A_4”). All of these were “absent” mutations found near regions with long stretches of Ns, as the participant reported 2223 and 8087 N’s for samples, respectively (i.e., somewhat of a dilution effect). We could not verify the results of the fourth participant for these duplicated samples.

### 3.3 Sequence interpretation

The dataset to calculate the self-reported Mutation Score was nearly complete–missing only from the two samples in rounds 3-5 where the virus was “not detected”, and not verifiable from one participant in rounds 3 and 4 that did not submit sequence files for the results. The mean mutation score was 1.5, with a median of 0.0 (range 0–13, s.d. 3.1) (Figure 2B). All four of the Mutation Scores higher than 4 were from a single participant in round 5 who apparently neglected to report mutations from the same region in each sample. The lineage was correctly reported 87 of 96 (91%) possible times. Of the 9 times it was incorrectly reported: 6 were from “not detected” results; two had too few data to assign a lineage, and one was from an apparent contamination.

## 4 Discussion

We analyzed the SARS-CoV-2 whole genome sequencing results from nine participants within Austria over five rounds of an EQA scheme. Our EQA scheme was designed to test two core competencies involved with SARS-CoV-2 sequencing: i) the ability to generate a consensus sequence from a sample and ii) the ability to interpret sequence data. The first competency deals with technical procedures involved with sample preparation (extraction, target enrichment/amplification, sequencing library preparation, analyzing raw sequencing data to prepare a consensus sequence). The second competency tests familiarity with sequence data: inspecting data, inferring coding regions and identifying mutations, and utilizing new resources to categorize the sequence (identify a lineage).

Other EQA schemes for SARS-CoV-2 sequencing have focused on the first competency, and also noted that consensus genomes were highly reproducible across platforms (Wegner et al., 2022). Our “Accuracy” Score could be called a “reproducibility” scoring system, as we used the consensus of all results for a given sample as the “true” sequence. Therefore, our data provide an indication about the accuracy of consensus genomes submitted to public sequence databases. From our dataset, over half of the submitted sequences contained at least 1 difference from the consensus (Figure 2A). The ultimate source(s) of these differences were unclear, and mostly seemed stochastic, as they were distributed throughout the genome. Some errors tended to appear at the end of sequencing gaps, suggesting strict post-sequencing quality control and bioinformatics should implemented–this was particularly true of the laboratory receiving accuracy scores >1 for the duplicated sample in round four (Figure 3, “D”). In general, most of the differences counted in the score were “absent” mutations–i.e., the consensus was different from the reference strain at that site but a mutation was not detected by the participant. Furthermore, we included a sample with known minor variants in round 4, which we assume was the result of a coinfection (Figure 3, “M”). The results were mixed (Supplementary Table), suggesting differences could be introduced during sample preparation and not necessarily bioinformatics steps. Notably, the results from this sample might be informative to standardizing NGS techniques for identifying HIV anti-drug mutations (Lee et al., 2020; Parkin et al., 2020). However, in some cases the same errors were reproduced within a laboratory in the same round, suggesting they may be occasionally systematic, e.g., introduced by primers and/or related to the bioinformatics pipeline (Figure 3).

As a second metric of the ability to generate a consensus sequence, we reported the sequence completeness. Nearly every laboratory could produce a 99% complete genome, independent of sample *C*_*t*_ value within the range of ∼20–31 *C*_*t*_ values (Figure 1). This highlights the usefulness of the tiled amplicon procedure for generating consensus genomes from patient samples (Quick et al., 2017; Tyson et al., 2020). All samples, particularly where coverage was less than 95%, showed a pattern of missing data consistent with amplicons generated for short-read sequencing (Figure 3). The fact that these errors were not associated with sample concentration indicates that these were due to errors in sample preparation (i.e., target enrichment steps or library preparation). Continued sequencing allows mutations that cause primer drop-out to be identified, and indeed tiled amplicon primer panels have undergone multiple versions as the SARS-CoV-2 virus has changed (Tyson et al., 2020). We noted some known primer dropout regions in the results associated with the ARTIC primers (Figure 3), but did not analyze whether other regions were associated with primer dropout, as we did not request participants submit raw sequence reads. As the target enrichment step relies on PCR, a key limitation of the approach is initial concentration of template in the test sample. However, we saw no relationship between genome completeness and estimated sample concentration (Figure 1A), and only a weak relationship between the percent of positive samples that were undetected per round and the estimated sample concentration (Figure 1B). We intentionally selected concentrated samples, and did not design a scheme to test the limit of detection. Others sequencing EQAs have included low concentration samples, and noted a significant reduction in the percent of completeness when sample concentration is diluted (Lau et al., 2022).

We observed additional, more serious, errors that were probably the result of pre-sequencing sample preparation steps. A participant in the first round - when variants contained fewer than 30 mutations per sample across the entire genome - reported mutations for two samples that were not included in the test panel, indicated laboratory contamination. Considering that most high throughput sequencing runs will include multiple SARS-CoV-2-positive samples, all with very similar genomes, cross-contamination remains difficult to detect. We tested this by including a negative sample in round 5 without informing the participants that one sample was negative. The sample was reported negative by two of the three participants. One participant stated that the sample “failed quality control” but submitted a partial sequence matching another sample in the panel and reported a lineage matching the same sample. It is crucial to maintain high standards to prevent and/or detect cross-contamination, particularly during post-PCR and pre-indexing library preparation steps.

The second competency that we evaluated was sequence interpretation. Viral variants can be detected and tracked with RT-qPCR techniques to identify SNPs based on melting curve analyses (Vogels et al., 2021). Interpreting these analyses requires additional competencies, as selecting the assays requires knowledge about circulating variants and familiarity with melting curve analyses (Camp et al., 2021; Buchta et al., 2022b). Whole genomes provide substantially more information and allow more specific identification of circulating variants. It was shown that laboratories that incorporated both RT-qPCR techniques and whole genome sequencing performed the best in terms of assigning lineages to a sample (Mögling et al., 2022). We found that identifying the lineage of a sequence was a relatively easy task for most laboratories that could generate a complete sequence from the sample. Similarly, “mistakes” in reporting mutations from the sequences could be explained by simple recording errors. Thus, in general, our observations indicated that errors in WGS results are likely from pre-sequencing sample preparation or post-sequencing consensus generation (bioinformatics). There were few errors in generating a lineage assignment or identifying mutations, regardless of the accuracy of the sequence.

**FIGURE 4:**
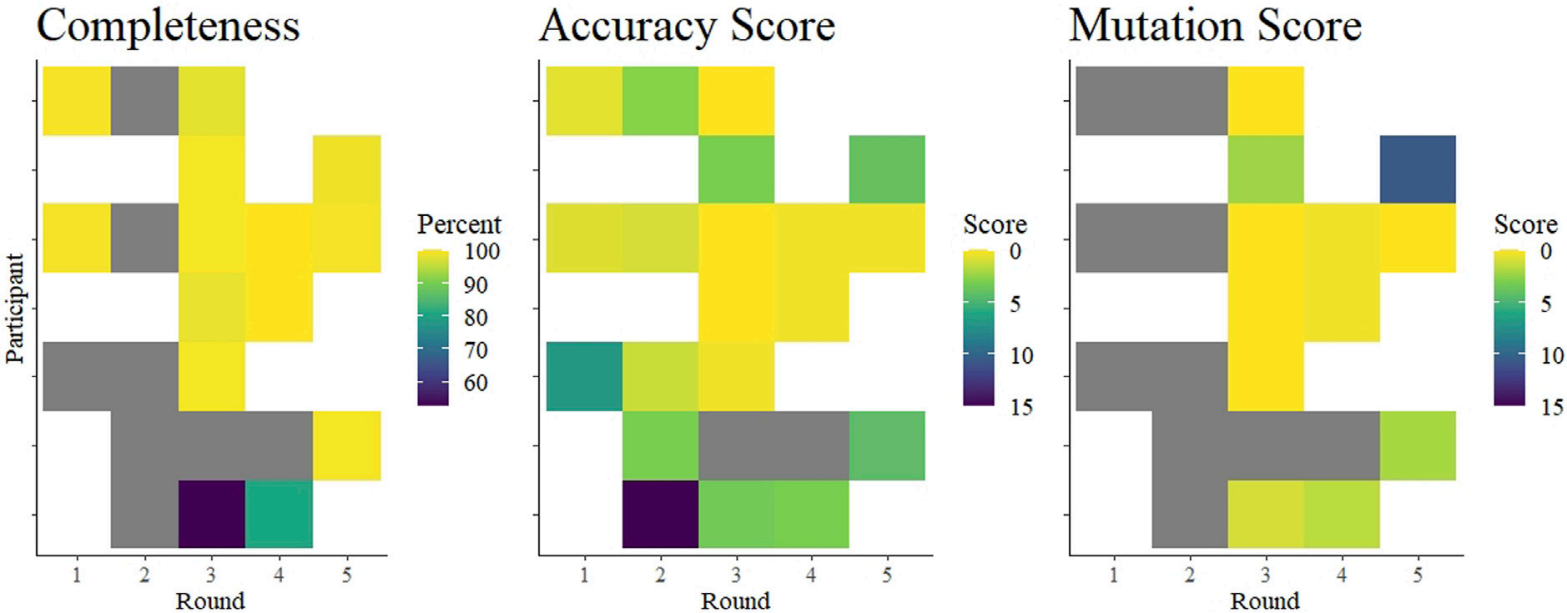
Summary of average completeness, accuracy score, and mutation score of each participant in each round. The colored tiles are shaded according to a gradient as indicated in each legend. White indicates no participation in that round. Gray squares indicate participation but data were missing. Specifically, accuracy scores could be interpreted from submitted data in rounds 1 and 2, but were not independently verifiable as no fastn sequences were submitted. Completeness scores from two laboratories in round 1 were based on submitted genome completeness information that was not specifically requested.

Surprisingly, we saw that overall performance did not change drastically over time (Figure 4). The mean values of percent completeness, accuracy score, and mutation scores remained similar across all rounds, although there were fewer outliers in the later rounds (Figure 2). Considering individual laboratories, some did show improvement over the course of the rounds in which they participated. As the reported protocols did not change (except for one participant), it seems that competency and familiarity with the procedures involved in NGS increased over time. NGS requires some proficiency with bioinformatics, and this competency remains the critical hurdle for laboratories beginning to implement NGS. This issue was ostensibly solved early in the pandemic by the availability of many services to process raw NGS reads and produce a consensus SARS-CoV-2 genome (O'Toole et al., 2021; Cheng et al., 2023; Hadfield et al., 2018). Nonetheless, interpreting the data and maintaining good quality control of the output requires trained personnel. As bioinformatics tools continue to develop, maintaining their open-source nature allows laboratories across the globe access to similar and reproducible analysis pipelines.

At the time of the pandemic, there were few external quality assessment schemes designed for next-generation sequencing in clinical virology. Prior to the COVID-19 pandemic, a well discussed example was the development of an emerging EQA scheme using NGS for HIV drug resistance testing (Parkin et al., 2020), and the issues concerning reproducibility and accuracy when using NGS in clinical virology were already appreciated (Avila-Rios et al., 2020). NGS data are complex, and represent challenges for the laboratory as well as for designing quality assessment schemes. Evaluating the quality of these data can focus on many facets–genome detection, sample preparation and sequencing, and bioinformatics–each of which could be evaluated separately. Our EQA scheme focused on generating consensus sequences from primary material, and had to evolve to accommodate the progressive accumulation of mutations in the SARS-CoV-2 genome. Namely, we changed the requirements to submit a consensus sequence instead of submitting all mutations. In retrospect, requesting a consensus sequence was essential to allow independent evaluation of the sequencing. Fundamentally, the consensus sequence represents the desired and reported outcome from WGS, similar to how detected/not detected is the fundamental reported outcome from virus genome detection assays. We did not request that participants provide quality metrics (e.g., average depth) or specifics about the analysis used to generate the consensus sequence, but these information would also be helpful in evaluating laboratory performance. However, such quality metrics are not requested commonly from virus detection EQA schemes (e.g., reporting specific Ct values, limit of detection, use of internal controls, etc.), and therefore we did not specifically request it. Others that requested participants report sequencing depth as part of their EQA scheme did not demonstrate any clear specific relationship between depth and other metrics (Lau et al., 2022; Wegner et al., 2022). Whether to request quality metrics from participants will depend on the fundamental goal of the EQA.

A principal limitation of this study is the fact that all laboratories did not participate in each round, and it is unknown whether registered participants intentionally did not participate because of “bad” results. Similarly, the small sample size prevents us from making comparisons between reagents, protocols and pipelines. However, these comparisons are probably better left to more controlled settings, where a single laboratory evaluates sample preparation methods across multiple samples (Charre et al., 2020; Liu et al., 2021) or bio-informatics pipelines with multiple datasets (Lee et al., 2020; SoRelle et al., 2020; de Vries et al., 2021; Krishnan et al., 2021). For viral WGS, EQA schemes are best suited to test two competencies, separately: sequencing and bioinformatics. By removing the bioinformatics portion, the ability to prepare a sample for sequencing and sequence the sample can be assessed while controlling for variability or deficiency in bioinformatics. Such deficiencies may be the reason for some of the seemingly random errors we observed (Figure 3). Similarly, a separate EQA scheme should test bioinformatics competency by supplying raw sequencing data and requesting a consensus sequence. This would eliminate the possibility of poor performance in sequence interpretation being due to problems with sequencing, and this is particularly important if sample degradation during shipping and handling is expected to be a source of error. The organization of bioinformatics schemes would rely on the availability of data from sequencing platforms in use and might only be feasible for ring tests with much larger enrollment. Such bioinformatics EQAs could also include evaluation of the applications of NGS–which we call “interpretation”–such as lineage assignment or inferring mutations. Others have also used EQA to measure applied competencies such as assigning samples to specific transmission clusters based on WGS data (Lau et al., 2022; Wegner et al., 2022). However, we observed very few errors in “interpretation” that could not be explained by simple data-entry/recording errors. Indeed, the most difficult aspect of organizing this EQA was designing the report form in a such a way that the requested data (fastn file, lineage assignment, and mutations) were correctly requested/reported. In the last round, there were still participants that could/did not follow the instructions, which we assume reflects the complexity of the analysis for the average clinical laboratorian.

Overall, we found that laboratories were prepared to implement next-generation sequencing methods to sequence whole genomes of SARS-CoV-2 relatively early in the COVID-19 pandemic. Excluding few outliers, participants achieved nearly 100% coverage of the genome, producing very nearly identical consensus sequences. Although we identified some deficiencies, we noted improvements within laboratories (Figure 4). As a caveat, these data may not be representative of the quality of sequences from Austria, as it is unknown whether the participants were performing routine sequencing with the reported protocols, or whether they were using the EQA to validate new protocols. Moreover, we know that not all Austrian laboratories submitting SARS-CoV-2 sequences to public databases participated in this EQA scheme. Nonetheless, our data suggest that the learning curve for implementing next-generation sequencing in a diagnostic laboratory is steep, but surmountable, and EQAs can help by providing independent feedback. These competencies are applicable towards achieving increased monitoring of seasonal virus epidemics, as well as enabling readiness for monitoring a future emerging zoonotic virus.

## Data Availability

The raw data supporting the conclusions of this article will be made available by the authors, without undue reservation.

## References

[B2] Angers-Loustau A. Petrillo M. Bengtsson-Palme J. Berendonk T. Blais B. Chan K. G. (2018). The challenges of designing a benchmark strategy for bioinformatics pipelines in the identification of antimicrobial resistance determinants using next generation sequencing technologies. F1000Res. F1000Res, ISCB Comm J-459. 10.12688/f1000research.14509.2 PMC6039958 30026930

[B3] Avila-Rios S. Parkin N. Swanstrom R. Paredes R. Shafer R. Ji H. (2020). Next-generation sequencing for HIV drug resistance testing: laboratory, clinical, and implementation considerations. Viruses 12 (6), 617. 10.3390/v12060617 32516949 PMC7354449

[B4] Buchta C. Aberle S. W. Allerberger F. Benka B. Gorzer I. Griesmacher A. (2023b). Performance of SARS-CoV-2 nucleic acid amplification testing in Austria as measured by external quality assessment schemes during 3 years of the COVID-19 pandemic: an observational retrospective study. Lancet Microbe 4, e1015–e1023. 10.1016/S2666-5247(23)00286-0 37979591

[B5] Buchta C. Camp J. V. Jovanovic J. Puchhammer-Stockl E. Strassl R. Muller M. M. (2022b). Results of a SARS-CoV-2 virus genome detection external quality assessment round focusing on sensitivity of assays and pooling of samples. Clin. Chem. Lab. Med. 60 (8), 1308–1312. 10.1515/cclm-2022-0263 35599330

[B6] Buchta C. Camp J. V. Jovanovic J. Radler U. Benka B. Puchhammer-Stockl E. (2022a). Inadequate design of mutation detection panels prevents interpretation of variants of concern: results of an external quality assessment for SARS-CoV-2 variant detection. Clin. Chem. Lab. Med. 60 (2), 291–298. 10.1515/cclm-2021-0889 34751522

[B8] Buchta C. Springer D. Jovanovic J. Borsodi C. Weidner L. Sareban N. (2023c). Three rounds of a national external quality assessment reveal a link between disharmonic anti-SARS-CoV-2 antibody quantifications and the infection stage. Clin. Chem. Lab. Med. 61 (7), 1349–1358. 10.1515/cclm-2022-1161 36756735

[B9] Buchta C. Zeichhardt H. Aberle S. W. Camp J. V. Gorzer I. Weseslindtner L. (2023a). Design of external quality assessment schemes and definition of the roles of their providers in future epidemics. Lancet Microbe 4 (7), e552–e562. 10.1016/S2666-5247(23)00072-1 37156257 PMC10162712

[B10] Camp J. V. Buchta C. Jovanovic J. Puchhammer-Stockl E. Benka B. Griesmacher A. (2021). RT-PCR based SARS-CoV-2 variant screening assays require careful quality control. J. Clin. Virol. 141, 104905. 10.1016/j.jcv.2021.104905 34273859 PMC8262392

[B11] Charre C. Ginevra C. Sabatier M. Regue H. Destras G. Brun S. (2020). Evaluation of NGS-based approaches for SARS-CoV-2 whole genome characterisation. Virus Evol. 6 (2), veaa075. 10.1093/ve/veaa075 33318859 PMC7665770

[B12] Cheng Y. Ji C. Zhou H. Y. Zheng H. Wu A. (2023). Web resources for SARS-CoV-2 genomic database, annotation, analysis and variant tracking. Viruses 15 (5), 1158. 10.3390/v15051158 37243244 PMC10222785

[B13] Corman V. M. Landt O. Kaiser M. Molenkamp R. Meijer A. Chu D. K. (2020). Detection of 2019 novel coronavirus (2019-nCoV) by real-time RT-PCR. Euro Surveill. 25 (3), 2000045. 10.2807/1560-7917.ES.2020.25.3.2000045 31992387 PMC6988269

[B14] de Vries J. J. C. Brown J. R. Fischer N. Sidorov I. A. Morfopoulou S. Huang J. (2021). Benchmark of thirteen bioinformatic pipelines for metagenomic virus diagnostics using datasets from clinical samples. J. Clin. Virol. 141, 104908. 10.1016/j.jcv.2021.104908 34273858 PMC7615111

[B15] Dudas G. Carvalho L. M. Bedford T. Tatem A. J. Baele G. Rambaut A. (2017). Virus genomes reveal factors that spread and sustained the Ebola epidemic. Nature 544 (7650), 309–315. 10.1038/nature22040 28405027 PMC5712493

[B16] Faria N. R. Quick J. Claro I. M. Theze J. de Jesus J. G. Pybus O. G. (2017). Establishment and cryptic transmission of Zika virus in Brazil and the Americas. Nature 546 (7658), 406–410. 10.1038/nature22401 28538727 PMC5722632

[B1] First NGS (2020). First NGS-based COVID-19 diagnostic. Nat. Biotechnol. 38 (7), 777. 10.1038/s41587-020-0608-y 32641848

[B17] Germer J. J. Abraham P. Mandrekar J. N. Yao J. D. (2013). Evaluation of the Abbott HBV RUO sequencing assay combined with laboratory-modified interpretive software. J. Clin. Microbiol. 51 (1), 95–100. 10.1128/JCM.02155-12 23100352 PMC3536212

[B18] Görzer I. Buchta C. Chiba P. Benka B. Camp J. V. Holzmann H. (2020). First results of a national external quality assessment scheme for the detection of SARS-CoV-2 genome sequences. J. Clin. Virol. 129, 104537. 10.1016/j.jcv.2020.104537 32659712 PMC7336937

[B19] Hadfield J. Megill C. Bell S. M. Huddleston J. Potter B. Callender C. (2018). Nextstrain: real-time tracking of pathogen evolution. Bioinformatics 34 (23), 4121–4123. 10.1093/bioinformatics/bty407 29790939 PMC6247931

[B20] Hanahoe H. Austin C. C. Shanahan H. (2021). Sharing COVID data? Check these recommendations and guidelines. Nature 592 (7855), 507. 10.1038/d41586-021-01028-5 33879885

[B21] Hodcroft E. B. De Maio N. Lanfear R. MacCannell D. R. Minh B. Q. Schmidt H. A. (2021). Want to track pandemic variants faster? Fix the bioinformatics bottleneck. Nature 591 (7848), 30–33. 10.1038/d41586-021-00525-x 33649511

[B22] Jennings L. J. Arcila M. E. Corless C. Kamel-Reid S. Lubin I. M. Pfeifer J. (2017). Guidelines for validation of next-generation sequencing-based oncology panels: a joint consensus recommendation of the association for molecular pathology and college of American pathologists. J. Mol. Diagn 19 (3), 341–365. 10.1016/j.jmoldx.2017.01.011 28341590 PMC6941185

[B23] Krishnan V. Utiramerur S. Ng Z. Datta S. Snyder M. P. Ashley E. A. (2021). Benchmarking workflows to assess performance and suitability of germline variant calling pipelines in clinical diagnostic assays. BMC Bioinforma. 22 (1), 85. 10.1186/s12859-020-03934-3 PMC7903625 33627090

[B24] Lau K. A. Horan K. Goncalves da Silva A. Kaufer A. Theis T. Ballard S. A. (2022). Proficiency testing for SARS-CoV-2 whole genome sequencing. Pathology 54 (5), 615–622. 10.1016/j.pathol.2022.04.002 35778290 PMC9239710

[B25] Lee E. R. Parkin N. Jennings C. Brumme C. J. Enns E. Casadella M. (2020). Performance comparison of next generation sequencing analysis pipelines for HIV-1 drug resistance testing. Sci. Rep. 10 (1), 1634. 10.1038/s41598-020-58544-z 32005884 PMC6994664

[B26] Liu T. Chen Z. Chen W. Chen X. Hosseini M. Yang Z. (2021). A benchmarking study of SARS-CoV-2 whole-genome sequencing protocols using COVID-19 patient samples. iScience 24 (8), 102892. 10.1016/j.isci.2021.102892 34308277 PMC8294598

[B27] Mögling R. Fischer C. Stanoeva K. R. Melidou A. Almeida Campos A. C. Drosten C. (2022). Sensitivity of detection and variant typing of SARS-CoV-2 in European laboratories. J. Clin. Microbiol. 60 (12), e0126122. 10.1128/jcm.01261-22 36445090 PMC9769866

[B28] O'Toole A. Scher E. Underwood A. Jackson B. Hill V. McCrone J. T. (2021). Assignment of epidemiological lineages in an emerging pandemic using the pangolin tool. Virus Evol. 7 (2), veab064. 10.1093/ve/veab064 34527285 PMC8344591

[B29] Oude Munnink B. B. Nieuwenhuijse D. F. Stein M. O'Toole A. Haverkate M. Mollers M. (2020). Dutch-Covid-19 response: rapid SARS-CoV-2 whole-genome sequencing and analysis for informed public health decision-making in The Netherlands. Nat. Med. 26 (9), 1405–1410. 10.1038/s41591-020-0997-y 32678356

[B30] Oude Munnink B. B. Worp N. Nieuwenhuijse D. F. Sikkema R. S. Haagmans B. Fouchier R. A. M. (2021). The next phase of SARS-CoV-2 surveillance: real-time molecular epidemiology. Nat. Med. 27 (9), 1518–1524. 10.1038/s41591-021-01472-w 34504335

[B31] Parkin N. T. Avila-Rios S. Bibby D. F. Brumme C. J. Eshleman S. H. Harrigan P. R. (2020). Multi-laboratory comparison of next-generation to sanger-based sequencing for HIV-1 drug resistance genotyping. Viruses 12 (7), 694. 10.3390/v12070694 32605062 PMC7411816

[B32] Quick J. (2020). nCoV-2019 sequencing protocol v3 (LoCost). protocols. 10.17504/protocols.io.bp2l6n26rgqe/v3

[B33] Quick J. Grubaugh N. D. Pullan S. T. Claro I. M. Smith A. D. Gangavarapu K. (2017). Multiplex PCR method for MinION and Illumina sequencing of Zika and other virus genomes directly from clinical samples. Nat. Protoc. 12 (6), 1261–1276. 10.1038/nprot.2017.066 28538739 PMC5902022

[B34] Reusken C. Broberg E. K. Haagmans B. Meijer A. Corman V. M. Papa A. (2020). Laboratory readiness and response for novel coronavirus (2019-nCoV) in expert laboratories in 30 EU/EEA countries, January 2020. Euro Surveill. 25 (6), 2000082. 10.2807/1560-7917.ES.2020.25.6.2000082 32046815 PMC7029448

[B35] Rossen J. W. A. Friedrich A. W. Moran-Gilad J. Genomic E. S. G. f. Molecular D. (2018). Practical issues in implementing whole-genome-sequencing in routine diagnostic microbiology. Clin. Microbiol. Infect. 24 (4), 355–360. 10.1016/j.cmi.2017.11.001 29117578

[B38] Shu Y. McCauley J. (2017). GISAID: global initiative on sharing all influenza data - from vision to reality. Euro Surveill. 22 (13), 30494. 10.2807/1560-7917.ES.2017.22.13.30494 28382917 PMC5388101

[B39] SoRelle J. A. Wachsmann M. Cantarel B. L. (2020). Assembling and validating bioinformatic pipelines for next-generation sequencing clinical assays. Arch. Pathol. Lab. Med. 144 (9), 1118–1130. 10.5858/arpa.2019-0476-RA 32045276

[B40] Tyson J. R. James P. Stoddart D. Sparks N. Wickenhagen A. Hall G. (2020). Improvements to the ARTIC multiplex PCR method for SARS-CoV-2 genome sequencing using nanopore. bioRxiv, 2020.09.04.283077. 10.1101/2020.09.04.283077

[B41] Vogels C. B. F. Breban M. I. Ott I. M. Alpert T. Petrone M. E. Watkins A. E. (2021). Multiplex qPCR discriminates variants of concern to enhance global surveillance of SARS-CoV-2. PLoS Biol. 19(5), e3001236. 10.1371/journal.pbio.3001236 33961632 PMC8133773

[B42] Wegner F. Roloff T. Huber M. Cordey S. Ramette A. Egli A. (2022). External quality assessment of SARS-CoV-2 sequencing: an ESGMD-SSM pilot trial across 15 European laboratories. J. Clin. Microbiol. 60 (1), e0169821. 10.1128/JCM.01698-21 34757834 PMC8769736

