# Supplementary material for: Virus sequencing performance during the SARS-CoV-2 pandemic: a retrospective analysis of data from multiple rounds of external quality assessment in Austria
Source: Front Mol Biosci. 2024 Feb 5;11:1327699. doi: 10.3389/fmolb.2024.1327699 (PMC10875003; doi:10.3389/fmolb.2024.1327699)
Supplement: Supplementary file 1 [file Table1.DOCX]

Supplementary Material

# Supplementary Figures and Tables

## Supplementary Figures


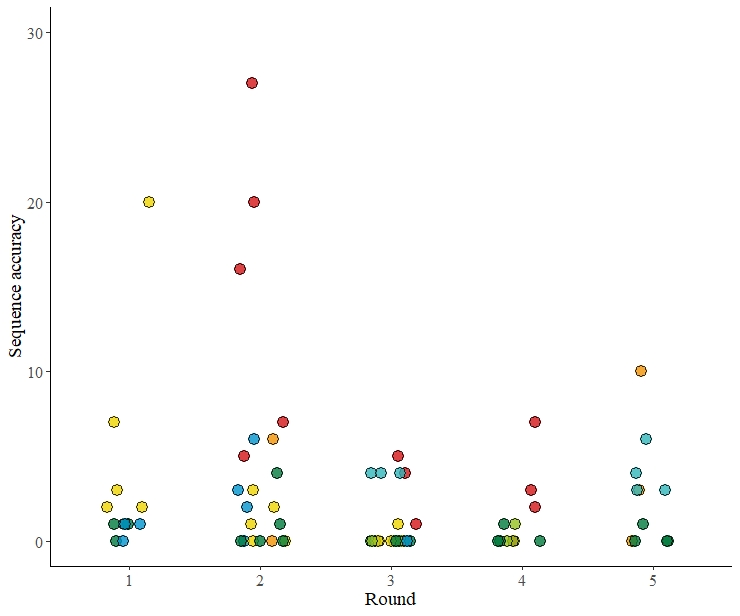


**Supplementary Figure 1.** Sequence accuracy score of each sample submitted over five rounds of SARS-CoV-2 sequencing EQA in Austria. Each point is colored uniquely for each participant to illustrate that outliers (high scores) tend to be from the same laboratory (e.g., yellow in rounds 1 and 2; red in rounds 2, 3 and 4; blue in rounds 2, 3 and 5).

## Supplementary Tables.

| **Site** | **Major Variant (%)*** | **Minor Variant (%)** | **Lab 1** | **Lab 2** | **Lab 3** | **Reference Lab** |
| --- | --- | --- | --- | --- | --- | --- |
| 22580 | A (66) | G (34) | N | G | G | A |
| 22629 | A (70) | C (30) | N | A | C | A |
| 22775 | A (89) | G (11) | N | A | A | A |
| 23073 | G (85) | A (15) | N | G | G | G |
| 28227 | C (63) | T (37) | C | C | C | C |

*Approximate, based on 20 000x average depth by the reference laboratory.

**Supplementary Table**. Mixed variants in round 4, sample 2, hCoV-19/Austria/MUW_1513519/2022, lineage BA.2. Shading indicates the majority variant.
